# Studying the organization of DNA repair by single-cell and single-molecule imaging

**DOI:** 10.1016/j.dnarep.2014.02.015

**Published:** 2014-08

**Authors:** Stephan Uphoff, Achillefs N. Kapanidis

**Affiliations:** a Department of Biochemistry, University of Oxford, Oxford OX1 3QU, United Kingdom; b Biological Physics Research Group, Clarendon Laboratory, Department of Physics, University of Oxford, Oxford OX1 3PU, United Kingdom

**Keywords:** DNA repair, DNA damage responses, Cell heterogeneity, Single-molecule fluorescence, Single-cell imaging, Super-resolution microscopy

## Abstract

DNA repair safeguards the genome against a diversity of DNA damaging agents. Although the mechanisms of many repair proteins have been examined separately *in vitro*, far less is known about the coordinated function of the whole repair machinery *in vivo*. Furthermore, single-cell studies indicate that DNA damage responses generate substantial variation in repair activities across cells. This review focuses on fluorescence imaging methods that offer a quantitative description of DNA repair in single cells by measuring protein concentrations, diffusion characteristics, localizations, interactions, and enzymatic rates. Emerging single-molecule and super-resolution microscopy methods now permit direct visualization of individual proteins and DNA repair events *in vivo*. We expect much can be learned about the organization of DNA repair by linking cell heterogeneity to mechanistic observations at the molecular level.

## Highlights

• Single-cell experiments to study stochastic events and heterogeneity in DNA repair.
• Quantifying DNA repair protein concentration, diffusion, and localization in cells.
• Direct observation of DNA repair using photoactivated single-molecule tracking.

## 1 Introduction: heterogeneity in DNA repair

DNA repair is responsible for preserving the genome of all cellular organisms. The DNA repair machinery also controls mutation rates that generate genetic variation in response to environmental changes. These conflicting tasks are finely tuned to achieve sufficient plasticity without compromising evolutionary stability. Mechanisms that create and control variation in DNA repair are therefore central to the survival of species. At the molecular level, heterogeneity is introduced by the stochastic occurrence of individual chemical reactions and molecule encounters. As a result, even seemingly homogeneous biological populations show differences between their individual components, such as variability in protein expression levels of clonal cells [1] or structural differences of molecules with identical chemical composition [2].

A central source of heterogeneity specific to DNA repair lies in the variety of exogenous and endogenous DNA damaging agents, ranging from UV light and radiation to alkylating chemicals and reactive oxygen species. The resulting diversity of toxic and mutagenic lesions favors a modular organization of repair in conserved pathways each involving a series of enzymes. Environmental changes cause further variation in the DNA repair system by triggering DNA damage and stress responses that induce specific repair proteins or activate large sets of genes through global regulatory networks. Cells thus face the difficult task of maintaining the proper balance of the entire repair system over a wide range of conditions to ensure that lesions are removed fast and accurately without accumulation of toxic intermediates [3]. Moreover, DNA repair needs to be coordinated with DNA replication, transcription, and chromosome organization processes, which can in turn be regulated by damage responses [4].

Although the main repair pathways have been identified and characterized using genetic, biochemical, biophysical, and cell biological approaches, we are still far from understanding the overall organization of DNA repair in cells. For instance, it is unclear how the different repair components cooperate to create functioning pathways, how the pathways coordinate and integrate with other cellular processes, and how environmental changes modulate the organization of the repair system. In short, a full systems-level description of DNA repair is needed, ideally based on the mechanisms of the individual enzymes. Many mysteries about DNA repair would be solved if the proteins could be directly observed in action inside single living cells, thus overcoming limitations inherent to measuring averages over large populations of molecules and cells. Building on single-cell microscopy, which has been performed for several hundred years, recent advances in instrumentation, biological labels and image analysis methods have orchestrated a shift in the way the data are analyzed with attention to quantitative evaluation of cell phenotypes and identifying the sources of their variation [5].

On the molecular scale, single-molecule methods have been very successful in unraveling many mechanisms of reconstituted DNA repair machinery *in vitro* (reviewed in this special issue). These approaches resolve molecular heterogeneity that cannot be separated biochemically, including repair complexes of different composition and stoichiometry as well as molecular subpopulations with different chemical modifications. Time-trajectories of individual molecules reveal dynamic heterogeneity, such as transient DNA-binding events, weak protein interactions, unsynchronized or stochastic enzymatic activities, and protein conformational dynamics [6]. The measurement conditions, however, are very different from the physiological environment, thereby raising the central question of how representative these results are for the function of molecules inside cells. Rendering single-molecule techniques compatible with *in vivo* observations is challenging due to the lack of control over intracellular conditions and difficulty of delivering probes in a non-invasive manner. Most progress on this front has been made using fluorescence microscopy in bacterial model systems. Hereafter, we discuss several example studies and hint at the challenges that need to be addressed in order to apply these techniques to study the organization of DNA repair *in vivo*.

## 2 Single-cell variation and DNA damage responses

**Fig. 1:**
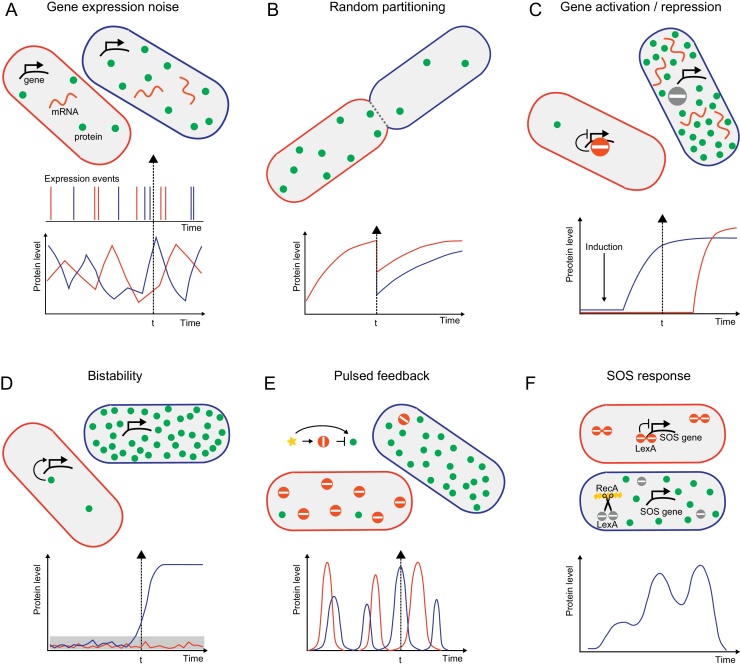
Sources of heterogeneous protein levels in genetically identical cells. Green circles represent a fluorescent reporter protein. (A) *Gene expression noise*: Protein levels fluctuate due to stochastic gene expression bursts and synthesis of multiple proteins per mRNA molecule. At time “*t*”, an example cell (red) has one mRNA copy and a low protein level, while the other cell (blue) has two mRNA copies and a higher protein level. (B) *Random partitioning*: Cell division occurs with different protein numbers in the two daughter cells. (C) *Gene activation/repression*: Following an induction signal, the timing of gene activation (or repression) is dictated by stochastic unbinding (or binding) events of a transcription repressor. (D) *Bistability*: Strong positive feedback regulation produces bistable behavior in which random excursions above an expression threshold trigger a complete switch in protein levels. (E) *Pulsed feedback*: Combination of positive and negative feedback regulation causes pulsed gene expression at varying amplitude and/or frequency. (F) *The SOS response is an example for a pulsed DNA damage response*: In the absence of damage, LexA dimers repress transcription of SOS genes. DNA damage triggers cleavage of LexA by active RecA, leading to pulses of SOS induction in a single cell (curve adapted from Ref. [18]).

We first consider the molecular origins of cell heterogeneity. Single-cell experiments uncovered variation in protein expression levels across cells and over time using genetically encoded fluorescent proteins. These studies showed that gene expression is a stochastic process and a fundamental source of cell individuality at the molecular level [1]. Even constitutively and highly expressed genes are subject to substantial noise due to bursts in transcription and protein synthesis from single mRNA transcripts [7] (Fig. 1A). Moreover, most mRNA and protein molecules partition randomly between daughter cells at cell division, which is expected to lead to significant variation [8] (Fig. 1B), particularly for DNA repair proteins with few copies per cell. Protein expression noise is further propagated through gene regulation networks. Random dissociation of a transcriptional repressor can cause drastic changes in protein expression [9] (Fig. 1C). Negative feedback aims to keep protein levels constant [10], but the precision of regulation is limited due to delays and information loss by stochastic transcription and translation events [11]. Positive feedback is prone to bistable behavior in which random fluctuations above an induction threshold lead to a switch in protein expression [12] (Fig. 1D).

DNA damage responses belong to a class of cellular decision-making processes [13], relying on accurately sensing inputs (*e.g.* the type and dose of damage, state in the cell cycle, nutrient availability) and translating them into appropriate outputs (*e.g.* repair, cell cycle arrest, adaptation, apoptosis) [14]. Cells can amplify specific repair activities upon damage in order to minimize the metabolic cost and mutagenic effects of repair in the absence of damage, but the flexibility puts the balance and coordination within and between repair pathways at risk. What strategies control the influence of noise on DNA repair systems? The combination of positive and negative feedback regulation provides a mechanism to regulate the strength and duration of a response [10] (Fig. 1E). For instance, the *Bacillus subtilis* energy stress response involving the alternative sigma factor *σ*^B^ is temporally modulated by stochastic pulses of gene activation with the strength of the response controlled by the frequency, not the magnitude of the pulses [15]. Stochastic fluctuations in the concentration of a phosphatase serve as the pulse trigger. Environmental stress, on the other hand, causes a single deterministic pulse with adaptive magnitude according to the rate of stress increase, thus serving as a temporal filter that allows cells to tune general and specific responses to the type and rate of environmental changes [16]. The mammalian DNA damage response by tumor suppressor p53 also occurs in oscillations or pulses that are shaped by feedback gene regulation and protein interactions [14].

Another example of controlling noise is the bacterial SOS response that counteracts double-strand breaks and replication-blocking lesions by inducing or repressing a large number of genes including those responsible for nucleotide excision repair, homologous recombination repair, mutagenesis, and inhibition of cell division. After cell variation in the SOS response was originally investigated using snapshots of *sulA* gene expression [17], subsequent time-resolved single-cell studies identified a precise pulse pattern of SOS induction on LexA-repressed promoters [18] (Fig. 1F). Pulses occurred at specific times after UV damage and their number increased with the amount of damage. Fluctuations in the inducing signal are dampened by LexA autoregulation to prevent false inductions and shorten the recovery time [19]. Simulations of the core SOS circuitry further highlighted the need to consider stochastic fluctuations for accurate descriptions of DNA damage responses [20].

Beyond heterogeneity introduced by stochastic molecular events, DNA damage can have different effects on cells at different stages in the cell cycle or during cell aging. For example, certain types of alkylation lesions block transcription and progression of replication forks, and therefore are less detrimental to cells in a stationary phase. Finally, DNA damage and repair cause mutations and genome rearrangements which irreversibly diversify cells in a clonal population. Taken together, single-cell experiments and theoretical studies suggest that stochastic events and cell variation play an important role in the organization and activity of the repair machinery.

## 3 Quantitative imaging of DNA repair processes

Building quantitative models of DNA repair is a difficult task because the determining factors, namely the reaction kinetics of the repair proteins, cannot be directly predicted from *in vitro* measurements due to their dependence on many other varying parameters: In the simplest view, the reaction kinetics are dictated by the enzymatic rates and the fluctuating concentrations of substrates and proteins in the cell. Importantly, the overall repair rate also includes the search time to locate a repair site, which itself depends on the protein mobility as well as the concentration, spatial distribution, and accessibility of the sites in the cell. These factors are further influenced by the local structure and global packaging of chromosomes. Substrate affinities and enzymatic rates may be enhanced by chemical modifications and cooperation with other repair factors that signal damage sites, or alternatively be repressed by competing enzymes in the case of overlapping, branching or conflicting repair pathways. We summarize quantitative fluorescence microscopy approaches that address these aspects, in particular the spatial distribution and movement of proteins in cells.

Assembly of repair proteins into complexes provides a mechanism to increase their local concentration at damage sites and orchestrate a series of reactions, as for replication forks [21] and transcription factories [22,23]. Eukaryotic cells exposed to ionizing radiation form repair foci where phosphorylation of histone H2AX in a 2-megabase region around a double-strand break is followed by accumulation of large numbers of repair factors [24]. Such clusters of Rad51 recombinase were first observed by fluorescence microscopy in immunostained fixed cells [25]; dynamic recruitment of homologous recombination repair proteins to sites of double-stranded breaks was later monitored using fluorescent protein fusions in live cells [26]. These and other studies established a spatial and temporal order of double-strand break repair involving chromosome reorganization, chromatin modifications, and assembly of DNA damage signaling and repair proteins. Bacterial double-strand break repair by homologous recombination also involves large structures of RecA (Rad51 homologue) [27–29], as discussed below. Toward establishing the sources and rates of spontaneous DNA breakage, a GFP fusion of the Gam protein was exploited as a label to directly count double-strand DNA breaks [30]. In the case of bacterial mismatch repair, long-lived foci of MutL proteins at sites of unrepaired mismatches served as markers for the emergence of mutations [31]; the number of foci increased by removing the proofreading function of the replicative DNA polymerase or the endonuclease step of the mismatch repair pathway. The inferred mutation rates appeared to be uniform across cells and correlated with the frequency of antibiotic resistant colonies. Dual-color imaging further showed the relative stoichiometry of MutL and MutS proteins in the same foci by their fluorescent intensities [32].

**Fig. 2:**
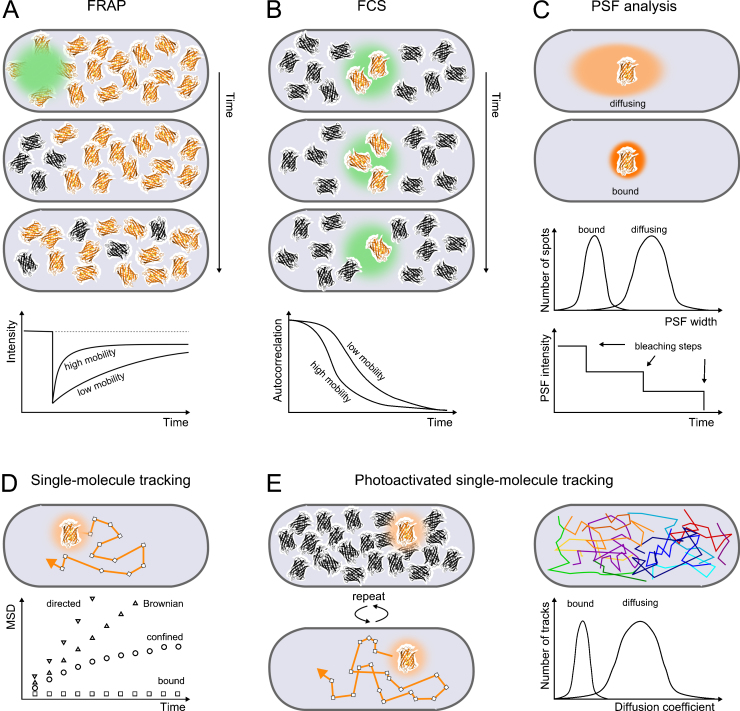
Methods for measuring protein mobility *in vivo*. (A) *FRAP*: The characteristic recovery time of the FRAP curve after bleaching reports on the protein mobility and exchange rate of molecules at binding sites within the bleaching spot. A difference between the pre- and post-bleach intensities indicates the presence of permanently bound molecules. (B) *FCS*: The decay time and amplitude of the autocorrelation curve report on the mobility and average concentration of proteins in the focus, respectively. (C) *PSF analysis*: The motion of a single molecule during the camera exposure time blurs the PSF. Histograms of the PSF width can be used to classify proteins of different mobility. The PSF intensity and sequential photobleaching steps report on the number of fluorescent molecules in a spot. (D) Single-molecule tracking connects localizations of one or few labeled molecules per cell to directly follow their motion. The mean squared displacement (MSD) as a function of the lag time between localizations summarizes the tracking data and distinguishes between immobile, confined, Brownian, or directed motion. (E) Photoactivated single-molecule tracking employs PALM to activate and track arbitrary numbers of labeled molecules per cell in a sequential manner. Protein mobility can be directly classified by the diffusion coefficient per track.

Transient repair activities in the absence of distinct foci can be probed by measuring protein mobility using Fluorescence Recovery After Photobleaching (FRAP); a method that monitors the replenishment of fluorescent proteins into a previously photobleached area inside single cells (Fig. 2A). Rapid fluorescence recovery shows high protein mobility whereas incomplete or slow recovery reports on the presence of DNA-bound molecules that exchange slowly with molecules outside the bleached area. This method revealed that mammalian nucleotide excision repair involves individual ERCC1 endonuclease molecules diffusing inside the nucleus and searching for randomly distributed repair sites [33]. ERCC1 joined repair complexes transiently upon UV exposure with a binding time of ∼4 min per single repair event. The repair initiation factor XPC, on the other hand, exhibited frequent short-lived binding events (∼300 ms) even in the absence of damage, which points at its role in detecting damage sites by scanning the genome using a combination of 3D and 1D diffusion [34]. Mathematical models were used to summarize these data, toward explaining the order and kinetics of repair complex assembly [35].

Alternatively, changes in protein mobilities can be measured on a confocal microscope using *in vivo* fluorescence correlation spectroscopy [36] (FCS; Fig. 2B). Diffusion of fluorescent molecules in and out of a focused laser spot results in stochastic intensity fluctuations. The mobility and concentration of molecules hence affects the time-scale of fluctuations as measured by the autocorrelation function of the intensity signal. FCS showed complex diffusion characteristics of cancer suppressor protein BRCA2 involving major populations of bound and slowly diffusing molecules and a minor mobile population; on the other hand, the diffusion of an unconjugated GFP control was consistent with a simple Brownian motion model [37]. DNA breaks triggered mobilization of the slow BRCA2 population, making it available for interaction with Rad51, as determined by simultaneously measuring FCS curves for both proteins using two distinct fluorophores. Joint fluctuations that appear in both fluorescence signals showed that BRCA2 and Rad51 diffuse together as complexes; their interaction could be quantified using cross-correlation analysis [36].

However, extracting quantitative information from FRAP and FCS curves requires precise knowledge of the laser focus size and geometry and works most reliably if the focus is placed inside a spatially homogenous sample, which limits the applicability to small bacteria whose dimensions are similar to the size of a diffraction-limited focus. Particular care has to be taken when analyzing heterogeneous populations of proteins due to ensemble averaging. The data fitting procedures with multiple free parameters and correction terms generally do not allow assigning a unique result for a mixture of diffusing populations or anomalous diffusion modes without making significant model assumptions [37,38]. These problems can be overcome by single-molecule imaging and tracking, as discussed below.

## 4 *In vivo* single-molecule and super-resolution microscopy

The action of individual molecules is crucial for the function of the whole cell [39]. For example, *Escherichia coli* replication in minimal growth medium involves just two forks per cell, with only few or single copies of a protein type per fork [21]. A single base modification can cause replication fork collapse [4] or manifest in a mutation that disrupts the function of a gene. In the event of a double-strand break, repair is performed by only ∼10 copies of RecBCD per cell and relies on finding the one particular homologous DNA sequence [40]. Yet, just a single unrepaired double-strand break can lead to complete chromosome degradation. The accurate performance of individual molecules at single repair sites can thus be crucial to cell viability. Understanding this relationship requires studying DNA repair at molecular resolution in live cells because each repair event occurs in a particular local environment of DNA structure, sequence and surrounding proteins. Moreover, such an undertaking provides direct information on the properties of the DNA repair machinery and facilitates the construction of quantitative models.

Fluorescence microscopy reaches single-molecule sensitivity by maximizing photon collection using high numerical aperture objectives, minimizing loss of signal with high-quality optical components, and reducing background noise through laser excitation tailored to the fluorophore spectrum and a small detection or excitation volume [2,6]. The latter is typically achieved using Total Internal Reflection Fluorescence (TIRF) microscopy with a laser beam that exits the microscope objective under a steep angle such that it gets reflected at the glass–water interface. This creates an evanescent excitation field with a depth of ∼100 nm and therefore limits excitation to fluorophores close to the surface of a microscope slide. Imaging deeper inside cells can be achieved using subcritical illumination angles to create a shallow excitation field [41]. In an alternative approach, light sheet illumination was recently adapted using an AFM cantilever mirror for detection of single transcription factors inside mammalian nuclei [42]. Isolated single molecules or complexes with a size below the optical resolution (a few hundred nanometers) appear as spots whose shape is dictated by the point-spread function (PSF) of the microscope. Analysis of the fluorescence intensity of a single PSF reveals the stoichiometry of proteins in foci [21,32,43], while its width reports on the diffusion characteristics because the PSF is blurred by molecule movement during the camera exposure time [44–46] (Fig. 2C).

The advent of super-resolution fluorescence microscopy [47] has opened new avenues for the study of DNA repair at the molecular scale inside cells, promising to uncover the architecture of repair complexes by direct observation. Performing confocal microscopy with two microscope objectives on either side of the sample increases the axial resolution for improved three-dimensional imaging. This technique, termed 4Pi microscopy, resolved a dense network of histone H2AX clusters and the spatial distribution of phosphorylated γ-H2AX foci following exposure to ionizing radiation [48]. Structured illumination microscopy (SIM) excites the sample with a series of grid patterns that push the resolution of the computationally reconstructed image approximately two-fold below the diffraction limit. SIM showed that BRCA1 and 53BP1 proteins occupy mutually exclusive volumes within such double-strand break repair foci, suggesting that their relative positioning directs the choice between homologous recombination or non-homologous end joining repair pathways [49]. A previously unobserved structure involved in bacterial double-strand break repair was discovered by extending SIM for imaging of live cells – following induction of a site-specific DNA break, homologous sequences on segregated sister chromosomes were paired after the formation of a thick bundle of RecA proteins in the space between the nucleoid and the cell membrane. Intriguingly, a thinner and highly mobile RecA filament was detected that extended into the interior of the nucleoid, presumably as part of the homology search process [29]. The increased resolution of SIM over conventional microscopy also helped to directly observe the spatial organization of eukaryotic meiotic chromosomes and revealed the ∼100 nanometer wide synaptonemal complex that is sandwiched between homologs [50].

Resolution down to a few tens of nanometers is achieved by Stochastic Optical Reconstruction Microscopy (STORM) [51] and Photoactivated Localization Microscopy (PALM) [52,53]. These methods employ photoswitching or photoactivation to image only a small subset of molecules at a time while the majority of fluorophores reside in a non-fluorescent state. Thus isolated emitters can then be localized with high precision by PSF fitting. The entire set of localizations acquired over the course of a movie forms the super-resolution image. This approach was applied to visualize telomere DNA loops that protect chromosome ends from DNA repair enzymes and prevent DNA damage signaling [54]. As in this case, most applications of localization microscopy have focused on crosslinked molecular structures *ex vivo*, fixed cells, or slow processes in live cells due to the requirement to record several thousand frames of blinking molecules to reconstruct a single image. Advanced localization analysis algorithms detect higher densities of molecules per frame [55,56], giving a prospect of imaging fast repair events and chromosome conformations in a series of reconstructed images. Recent developments in camera and image processing technologies increased STORM acquisition rates up to 32 reconstructed images per second [57]. However, very high laser powers are required to achieve fast photoswitching and bright PSFs, raising concerns about phototoxicity in live cell experiments, especially for the DNA repair field.

Beyond super-resolution microscopy of cellular structures, PALM can be extended to follow the movement of individual molecules in live cells [58]. Previously, single-particle tracking techniques that connect localizations in a series of images to form trajectories were restricted to low particle densities (Fig. 2D). PALM enables tracking almost arbitrary numbers of molecules by sequential photoactivation (Fig. 2E). Measuring relatively slow diffusion of molecules within the cell membrane has been the main target of single-particle tracking techniques, benefiting from the two-dimensional structure of the membrane and its accessibility for labeling [59]. Increased sensitivity and temporal resolution of fluorescence microscopes allowed tracking individual diffusing fluorescent proteins in the bacterial cytoplasm [60], hence opening the technique for application to any bacterial fusion protein. Single-particle tracking can also be used to identify the most appropriate model for FRAP and FCS analysis [61]. Applying this approach to p53 yielded a consistent set of estimates for the chromatin-bound fraction and binding times for the three complementary methods [61].

Furthermore, PALM provides a direct way of counting molecules in cells [62–64]; a promising feature for the study of protein complex stoichiometry and quantitative description of reaction networks [46]. Using photoactivatable fluorescent proteins that bleach irreversibly, each activation event can be counted as one protein. Nonetheless, obtaining precise and absolute numbers is hindered by fluorophore maturation and blinking, as well as non-uniform excitation, detection, and photobleaching conditions [22,62–64]. Further development of fluorescent proteins with improved photophysical characteristics and establishing standardized calibration routines will be crucial for accurate molecule counting.

## 5 Direct observation and quantification of bacterial base-excision repair

We recently reported imaging individual base-excision repair events in live *E. coli* cells using photoactivated single-molecule tracking [46]. DNA polymerase I (Pol1) and DNA ligase belong to conserved protein families that perform repair synthesis and ligation in excision repair pathways as well as lagging strand replication. *In vitro* single-molecule studies elucidated how Pol1 binds DNA primers [65], distinguishes correct from incorrect nucleotides [66], and performs DNA synthesis [67]. However, these results could only hint at the actual *in vivo* reaction kinetics, calling for live-cell measurements to investigate the spatial organization and coordination of Pol1 and Ligase in reaction pathways.

**Fig. 3:**
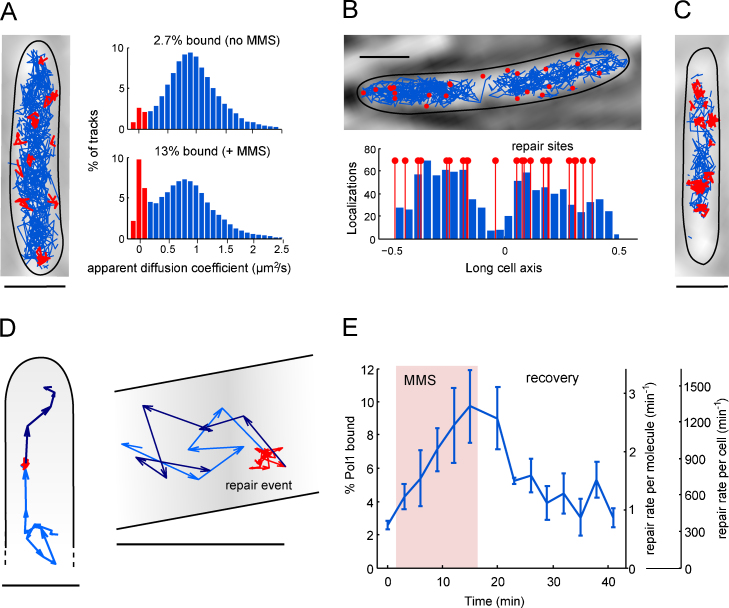
Measuring single base-excision repair events by DNA polymerase I (Pol1) in live *E. coli*. Scale bars: 1 μm. Figures adapted from [46]. (A) Photoactivated single-molecule tracking gives a map of Pol1 tracks in a cell with DNA damage by methyl methanesulfonate (MMS). Based on the mean squared displacement (MSD), individual tracks with a low apparent diffusion coefficient are shown in red while tracks of freely diffusing Pol1 are shown in blue. Histograms of the apparent diffusion coefficient can be used to quantify the fraction of bound Pol1 molecules in the absence and presence of MMS damage. (B) The localizations of bound Pol1 molecules (red dots) show that base-excision repair sites are randomly positioned throughout the nucleoid. The histogram shows the distribution of unbound Pol1 molecules across the long cells axis; positions of bound molecules are shown in red. (C) Prolonged treatment with a low dose of MMS for 1 h causes chromosome compaction, as evident from the confinement of tracks to a smaller area compared to cells in panels A and B that were imaged within 20 min of MMS treatment. (D) Individual Pol1 tracks display the search path to find a repair site (light blue), a complete repair event (red), and further diffusion (dark blue). (E) Quantifying the Pol1 damage response during 15 min MMS treatment and subsequent recovery. Using the binding time per repair event and the percentage of bound molecules gives the repair rate per Pol1 molecule. The total repair rate per cell is estimated by counting the number of Pol1 tracks per cell.

Fusions of Pol1 and Ligase with photoactivatable fluorescent protein PAmCherry [68] were expressed from their native chromosomal locations, ensuring wild-type expression levels. Tracks of single activated molecules displayed rapid diffusion in undamaged cells (Movie 1), whereas DNA damage by methyl-methanesulfonate (MMS) caused transient immobilization of single molecules (Fig. 3A, Movie 2) [69]. Similar to mammalian nucleotide-excision repair [33], the individual reaction sites were distributed throughout the nucleoid (Fig. 3B). Simulations confirmed that Pol1 and Ligase did not form larger repair complexes in the cytoplasm but performed Brownian motion as individual molecules.

Pol1 and Ligase tracks recapitulated the organization of the chromosome into ellipsoidal lobes [46]. Prolonged DNA damage caused chromosome condensation (Fig. 3C), a strategy cells could employ to sequester the genome from damaging agents [70]. The nucleoid-association of Pol1 and Ligase suggests short-lived non-specific protein–DNA interactions that might be part of a facilitated diffusion process to search for lesions. *In vitro* studies showed sliding on stretched DNA for a range of DNA-binding and repair proteins including p53 [71], MutS/MutL [72], oxoguanine glycosylase [73], and UvrAB nucleotide excision repair complexes [74]. On the other hand, *E. coli* RNA polymerase appeared to encounter promoters by direct collision without significant sliding [75]. This might be the ideal search mechanism in the case of high intracellular protein concentrations [75] and enforces the question whether facilitated diffusion actually plays a role for most DNA-binding proteins *in vivo*. For lac repressor tetramers, present at about five copies per cell, facilitated diffusion does occur in *E. coli*, confirmed by measuring single-molecule association rates on pairs of operator sites in close proximity [76].

Pol1 and Ligase tracks showed several examples of entire repair cycles including the diffusion trajectory to locate a lesion, transient binding for DNA repair, and continued diffusion [46] (Fig. 3D). Using a combination of PALM, single-molecule tracking, and PSF width measurements, the distribution of binding times to repair a single DNA gap or nick could be determined, yielding average values of 2.1 s for Pol1 and 2.5 s for Ligase. Counting Pol1 and Ligase tracks and quantifying the fraction of DNA-bound and freely diffusing molecules provided a direct readout in a single measurement of the repair rates and search times at the single-cell level, over time, and for varying MMS concentrations. Repair rates of Pol1 and Ligase increased within minutes of exposure to MMS (Fig. 3E). Yet, the majority of molecules remained unbound even at high MMS concentrations, thereby saturating gapped and nicked DNA substrates. The overall base-excision repair activity was limited by upstream steps in the pathway, ensuring minimal presence of unbound DNA breaks and high repair capacity to prevent accumulation of DNA repair intermediates. We expect the combination of single-molecule and single-cell analysis [77] will prove very useful for the DNA repair field toward establishing a systems-level description of repair pathways based on mechanistic observations of molecular processes in live cells.

## 6 Future directions

Key advances to aid live-cell single-molecule imaging, localization microscopy, and molecule counting include the creation of more photostable and brighter fluorescent proteins as well as understanding and controlling their *in vivo* photophysics. Promising strategies are under development for labeling intracellular proteins with bright synthetic photoswitchable fluorophores [78], or using electroporation to internalize purified and labeled proteins [79]. Detailed mechanistic descriptions might be brought by structural studies on single enzymes *in vivo*. Single-molecule Förster Resonance Energy Transfer (FRET) provides information on intra- and intermolecular distances with millisecond temporal resolution [2,6]. Toward measuring single-molecule FRET *in vivo*, purified and labeled proteins were inserted into the cell membrane or internalized by microinjection [80,81], but these approaches are not well suited to investigate nuclear or bacterial repair proteins. The combination of photoswitching and single-molecule FRET is a step toward super-resolution FRET microscopy to resolve high concentrations of molecules and their FRET states by photoswitching [82]. Current microscopy techniques have been designed for the purpose of observation with minimal perturbation to the system; yet, understanding molecular mechanisms of repair beyond mere observation requires measuring responses upon specific genetic, biochemical, or mechanical perturbations. Efforts in this direction can build on achievements in optogenetics [83], nano-manipulation technologies [84,85], and microfluidics [86].

The methods and pioneering studies discussed here show that a molecular description of DNA repair as it occurs in single cells is within reach. The next challenge will be the integration of these observations to uncover the fundamental organization principles of DNA repair.

## Conflict of interest

The authors declare there are no conflicts of interest.

## Appendix A Supplementary data

Movie 1 The video shows a section of a PALM movie with a single photoactivated Pol1-PAmCherry fusion protein diffusing inside a live *E. coli* cell. The fluorescence intensity data (blue color map) is overlaid on the transmitted light microscopy image showing the cell outline (grey scale image). Reproduced from Ref. [46] with permission from PNAS.

Movie 2 The video shows a section of a PALM movie similar to Movie 1, but for *E. coli* cells under DNA damage treatment with MMS. The stationary fluorescence spots correspond to Pol1 molecules performing transient DNA repair reactions. Reproduced from Ref. [46] with permission from PNAS.
