# Supplementary figures and images for: Studying the organization of DNA repair by single-cell and single-molecule imaging
Source: DNA Repair (Amst). 2014 Aug;20(100):32–40. doi: 10.1016/j.dnarep.2014.02.015 (PMC4119245; doi:10.1016/j.dnarep.2014.02.015)

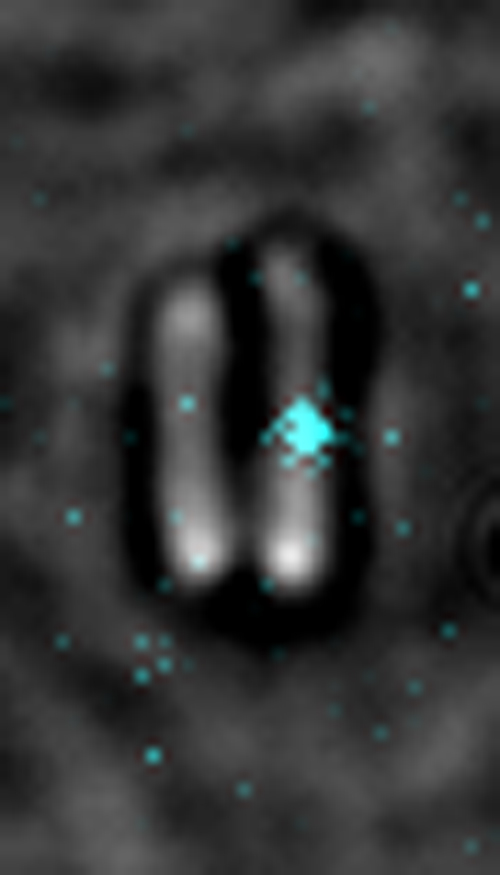

Supplement: Movie 1 — The video shows a section of a PALM movie with a single photoactivated Pol1-PAmCherry fusion protein diffusing inside a live E. coli cell. The fluorescence intensity data (blue color map) is overlaid on the transmitted light microscopy image showing the cell outline (grey scale image). Reproduced from Ref. [46] with permission from PNAS. [file mmc1.jpg]

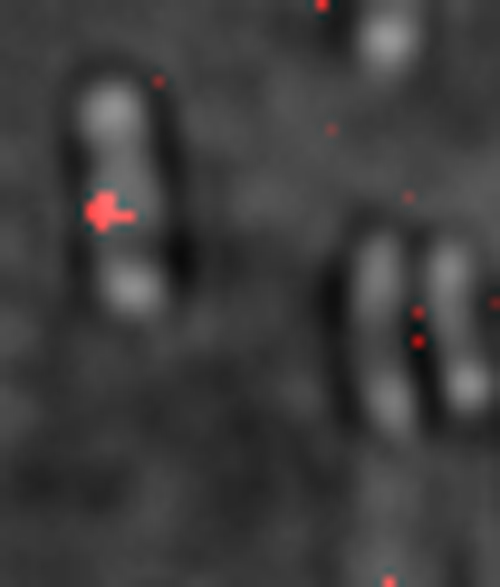

Supplement: Movie 2 — The video shows a section of a PALM movie similar to Movie 1, but for E. coli cells under DNA damage treatment with MMS. The stationary fluorescence spots correspond to Pol1 molecules performing transient DNA repair reactions. Reproduced from Ref. [46] with permission from PNAS. [file mmc2.jpg]
